# AcMNPV, a viral insecticide of Lepidoptera pests, stimulates the immune response of the natural enemy *Arma chinensis* Fallou

**DOI:** 10.1128/aem.00613-25

**Published:** 2025-05-27

**Authors:** Ximei Yuan, Qingyao Meng, Zhengjie Lu, Chen Wang, Yiqing He, Ke Li, Juan Du, Yuejun Fu

**Affiliations:** 1 Key Laboratory of Chemical Biology and Molecular Engineering of Ministry of Education, Shanxi Key Laboratory of Biotechnology, Institute of Biotechnology, Shanxi University 631296 , Taiyuan, China; 2 Zhejiang Academy of Science & Technology for Inspection & Quarantine, Hangzhou, China; UMR Processus Infectieux en Milieu Insulaire Tropical, Ste. Clotilde, France

**Keywords:** AcMNPV, miR-8, *Arma chinensis *Fallou, Toll receptor, Defensin

## Abstract

*Autographa Californica* multiple nucleopolyhedrovirus (AcMNPV) is the most widely studied baculovirus currently used as a biopesticide application for crop pest control. AcMNPV has a good control effect on Lepidoptera pests, but the impact of AcMNPV on other insects in the environment, especially natural enemies, is worth further discussion. *Arma chinensis* Fallou, an essential natural enemy insect widely distributed in China, can prey on multiple pests that cause severe damage to the production economy and can play an effective role in preventing agroforestry pests. In this study, the natural enemy *Arma chinensis* Fallou was used as the research material to analyze the effect of AcMNPV on the immune system of *Arma chinensis* Fallou. The immune mechanism of AcMNPV-EGFP in infecting *A. chinensis* was revealed for the first time, and the critical factor miR-8 was found. The relationship between AcMNPV, miR-8, and *A. chinensis* was described. RNAi pathway and JAK/STAT signaling pathway are the main antiviral pathways in insects, and we have newly discovered the molecular regulation mechanism of the Toll signaling pathway in antiviral immunity. In a word, AcMNPV, a Lepidopteran insecticide, affects the immune response of *Arma chinensis* Fallou and has side effects on natural enemies in the process of pest control.

**IMPORTANCE:**

AcMNPV is a viral pesticide that is widely used to control Lepidoptera pests. However, the impact on other insects has yet to be thoroughly studied. Therefore, the toxicity and side effects of AcMNPV on non-target organisms need to be effectively evaluated. As a biological control agent in agricultural ecosystems, natural enemy insects can effectively control the number of pests and reduce crop damage. Compared with chemical pesticides, natural enemy insects can avoid or reduce pesticide residues in agricultural products and have no environmental pollution, and pests do not quickly develop resistance to them. It will help ensure crops’ safe production and balance the ecosystem. In this study, AcMNPV-EGFP was used to infect the natural enemy-*Arma chinensis* Fallou, which proved the effect of the virus pesticide on *Arma chinensis* Fallou. This work helps optimize the joint use of the natural enemy-*Arma chinensis* Fallou and other insecticides in applying biological control and also provides a reference for understanding the mechanism of insect antiviral infection.

## INTRODUCTION

Many investigations show that agricultural and forestry pests have developed apparent resistance to chemical pesticides, and the extensive use of these chemicals will damage the ecological environment. Using the original species or biological insecticides in nature to control pests can effectively reduce environmental pollution and ecological damage. Baculovirus is a variety of viruses with a double-stranded circular supercoiled genome. There are two types of virions: occlusion-derived virus (ODV) and budding virus (BV) (1–3). Baculovirus is widely used as a biological insecticide in crop pest control and an efficient eukaryotic expression vector of exogenous genes. It has the following advantages: it specifically infects arthropods (1) and is not toxic to vertebrates, so it is safe for the human body. Compared with chemical pesticides, it does not cause environmental pollution and has low risk; Baculovirus can also be used with other control methods. AcMNPV is a model species of baculovirus. Its genome is about 134 kbp, encoding 154 open reading frames (ORFs) (3). AcMNPV and its recombinant virus have a good control effect on Lepidopteran pests, but the virus’s safety to other insects needs to be further evaluated.

*Arma chinensis* Fallou (Hemiptera: Pentatomidae) is an essential natural enemy insect, which can prey on a variety of pests that cause serious damage to the production economy and has a good control effect on agricultural and forestry pests (4). However, *A. chinensis* is susceptible to microbial infection and insecticides during its development, resulting in death. Therefore, studying the innate immune pathway and the mechanism of development and reproduction of *A. chinensis* will help promote its field application of *A. chinensis* and improve its damage control ability. Although AcMNPV is a unique virus in Lepidoptera, many studies have shown that AcMNPV can infect non-host insects (5–7). Therefore, the study on the effect of AcMNPV on the immunity of *A. chinensis* and the molecular mechanism of its interaction will help to promote the field application of *A. chinensis* and optimize the joint application with biological insecticides.

Insects have an immune response system that can respond quickly to pathogen infection, including cellular immunity and humoral immunity. The humoral immune response is mainly the production of antimicrobial peptides (AMPs) and melanization coating reaction (8), in which the expression of AMPs is mainly regulated by Toll and IMD signaling pathways, which are stimulated by bacteria and fungi, and JAK/STAT signaling pathway is mainly stimulated by virus infection (9–12). Although JAK/STAT plays a crucial role in immune regulation against different viral infections, the Toll signaling pathway also has potential functions in insect antiviral immunity. AMP is a class of small alkaline peptides with a positive charge (13). Because insect AMPs can inhibit the synthesis of the cell wall of pathogenic bacteria, inhibit the expression and reproduction of their genes by binding with virions, and lead to the leakage of cell contents by acting with negative charge groups on the surface of parasitic cells, or combine with phospholipids on the cell membrane to induce the permeability of tumor cell membrane to dissolve them (13–16), AMPs have the effects of inhibiting bacteria, anticancer, antiviral, and anti-inflammatory diseases and are not easy to generate drug resistance (17). Therefore, antimicrobial peptides have great application value in medical treatment.

MicroRNA (miRNA) is a class of single-stranded RNA molecules encoded in organisms, with a length of about 21–23 nt. miRNA can target multiple mRNAs and inhibit the translation of target mRNA by complementing and pairing with the 3′UTR of target gene mRNA (18–20). Since miRNA regulates the expression of target genes at the post-transcriptional level, it must play an important role in maintaining insect immune homeostasis. miR-8 widely exists in bilaterally symmetrical animals (21) and is highly conserved from nematodes to humans (22). *Toll* and *Dorsal* of the Toll signaling pathway in Drosophila are targets of miR-8. In the nonimmune state, the decrease of miR-8 leads to the increase of AMP, but ectopic melanization occurs in Drosophila with the deletion of miR-8 mutation, which is lethal, indicating that miR-8 can prevent abnormal activation of immune activity by inhibiting multiple immune genes, thus protecting the insect from potential damage caused by chronic immune activation (23). In addition, miR-8 of *Bombyx mori* can downregulate the expression of *ie1* and *vp39* genes of BmNPV and inhibit the replication of the BmNPV genome (24). These studies indicate that miR-8 may be involved in the process of insect antimicrobial.

Lepidopteran pests seriously threaten agroforestry development, and AcMNPV is widely used as an insecticide. What happens to other organisms when AcMNPV is used for pest control? *A. chinensis*, as a natural enemy, preys on Lepidopteran pests. Investigating the effects of AcMNPV on *A. chinensis*’s immune system and the host’s molecular mechanisms of resistance to AcMNPV during predation is crucial. At present, it has been confirmed that *Serpin*, *Toll*, *Drosal*, and *Ush* of miR-8 targets and its predictive targets GNBP3 and PvF are involved in the immune mechanism of insect resistance to microorganisms and regulate the expression of AMPs through signaling pathways (21, 23, 25). miR-8 can target the critical genes of the virus to inhibit virus proliferation. Therefore, this project focuses on and analyzes the response molecular mechanism of miR-8 in AcMNPV-EGFP infecting *A. chinensis*. It provides the experimental basis for further protecting natural enemy insects, using them to carry out biological control and combined use with insecticides.

## MATERIALS AND METHODS

### Cell lines, viruses, and insects

*Spodoptera frugiperda* IPLB-Sf21-AE clonal isolate 9 (Sf9) insect cells were cultured in SIM SF serum-free insect SF9/SF21 cell medium (Sino Biological Inc., Bei-jing, China) in a 27°C cell incubator. Recombinant baculovirus AcMNPV-EGFP, constructed by inserting the *egfp* gene in the *p10* promoter locus of AcMNPV, was used for infection of Sf9 cells. Eggs of *A. chinensis* (Baiyun Industry Co., Ltd., Henan, China) were maintained in 12-well microtiter plates with *Tenebrio molitor* pupae, under 26°C ± 2°C, 70%–80% relative humidity, and natural photoperiod.

### Injection of larvae with miRNA and AcMNPV-EFGP

miR-8 mimics (5′-UAAUACUGUCAGGUAAAGAUGUC-3′) and miR-8 inhibitor (5′-GACAUCUUUACCUGACAGUAUUA-3′) were chemically synthesized and modified by Sangon Biotechnology Corp (Shanghai, China). miR-8 mimics was an unmodified RNA oligo with the same sequence as miR-8. The miR-8 inhibitor was complementary to miR-8 with 21-*O*-methyl modification in the sugar. The negative control of mimics (5′-UUGUACUACACAAAAGUACUG-3′, mimics NC) and inhibitor (5′-CAGUACUUUUGUGUACAA-3′, inhibitor NC) were provided by Sangon Biotechnology Corp. The mimics, inhibitor, and NC were used to inject the 4th instar *A. chinensis* larvae. The larvae were infected with 2.6 × 10^13^ vp/μL and 2.8 × 10^9^ vp/μL AcMNPV-EGFP′ BVs separately at 8 h after injection with 10 nM miR-8 mimics, miR-8 inhibitor, miR-8 mimics NC, and miR-8 inhibitor NC, respectively.

### Bioinformatic analyses

*Toll* and *AMP* genes were identified from the transcriptome of *A. chinensis* (GeneBank accession number SRX6830878). The open reading frame (ORF) sequences of these genes were obtained from the online website (https://emboss.bioinformatics.nl/cgi-bin/emboss/getorf). ExPASy Molecular Biology Server (https://www.expasy.org/) predicts molecular weight and theoretical isoelectric point. GSDs 2.0 (http://gsds.gao-lab.org/) was used to predict the exon-intron organization of these ORFs. Multiple sequence alignments were generated using Cluster X2 sequence analysis software. We used the NCBI CD-search tool to predict conservative domains (https://www.ncbi.nlm.nih.gov/Structure/cdd/wrpsb.cgi). The phylogenetic tree was constructed using the Neighbor-Wing algorithm (1,000 repetitions) and MEGA 11 software (26).

### dsRNA synthesis

T7 promoter sequence was added to specific primers, and DNA templates for dsRNA synthesis were amplified by PCR, followed by dsRNA production with the TranscriptAid T7 High Yield Transcription Kit, after which dsRNA purification was performed. The primer sequences are shown in Table 1.

**TABLE 1:** Sequences of primers

Primer	Sequence (5′ → 3′)
Primers for qPCR analysis	
*β-actin* F	AAGGCTAACCGTGAGAAGATGAC
*β-actin* R	GATTGGGACAGTGTGGGAGAC
*5S rRNA* F	GCCAACGTCCATACCATGTT
*5S rRNA* R	GTGGTGTTCCCAGGCGGTCA
*miR-8* F	ACACTCCAGCTGGGTAATACTGTCAGGTAAA
*miR-8* R	CTCAACTGGTGTCGTGGAGTCGGCAATTCAGTTGAGGACATCTT
*Toll* F	GCCCTGGAACAGCAGATTG
*Toll* R	TGATTTGGATGGACGGTAG
*Toll4* F	ACTGGTGGCTGTGGTGGTC
*Toll4* R	AAGCGTCCGAAGGGAACAT
*Toll6-1* F	AGACTACCAGCAGCAGAGG
*Toll6-1* R	TTATACGAAATAGGTCCTTCC
*Toll6* F	GCTGCTCTGGCTCTGGAAT
*Toll6* R	CGTAGCGAAGGGTGGTGTT
*Toll7* F	CTGGTGACTTTCCCTGTT
*Toll7* R	GTCCGTGTCCGATACTTT
*Tollo* F	GTCGCTAGGTAGGCTACTTGAGATG
*Tollo* R	CACGAGTATCGGTGAATATCTCTGG
*Defensin-1* F	CCACGGCCCTTCCTGTCTTCGAAG
*Defensin-1* R	AATTCATCAGCGACAGGTGCTTCT
*Defensin-2* F	CTCATCATCTTCGCTGCTG
*Defensin-2* R	ATATTCATCATTCTCTTGGAGTTCC
*Ac-ie1* F	GGAATCCCTTGAGCAGCCTGTTG
*Ac-ie1* R	AGTTGCCGATGGTTGGTTCACAC
*Ac-vp39* F	TTGCGCAACGACTTTATACC
*Ac-vp39* R	CGGCGGCACGGGAACACATTTTAG
Primers for dsRNA synthesis	
ds*Toll* F	TAATACGACTCACTATAGGGCTGGGTCGTAGGCGAATTTA
ds*Toll* R	TAATACGACTCACTATAGGGGTAATGGCCGGAGGTGTAGA
ds*Toll4* F	TAATACGACTCACTATAGGGCACTCGCTTCGAGACCTTTC
ds*Toll4* R	TAATACGACTCACTATAGGGATACTGCGCTCAGGTCCAGT
ds*Toll6-1* F	TAATACGACTCACTATAGGGTTATGGGTCCATTCCCGTTA
ds*Toll6-1* R	TAATACGACTCACTATAGGGCCTTCAGCGGTCTTTCTCTG
ds*Toll6* F	TAATACGACTCACTATAGGGCTCAGGGAATTGGATCTGGA
ds*Toll6* R	TAATACGACTCACTATAGGGGGTGTCTAAGTCCAGCGAGC
ds*Toll7* F	TAATACGACTCACTATAGGGCCGAGCAATTTGAAGTGGTT
ds*Toll7* R	TAATACGACTCACTATAGGGTCTTCAGATAAGGCCGGAGA
ds*Tollo* F	TAATACGACTCACTATAGGGATGACCTGCCCTACCAACTG
ds*Tollo* R	TAATACGACTCACTATAGGGCAAGGGTTGACTCCAATGCT
ds*EGFP* F	TAATACGACTCACTATAGGGCCTCGTGACCACCCTGACCTAC
ds*EGFP* R	TAATACGACTCACTATAGGGTTGCCGTCGTCCTTGAAGAAGATG

### Injection of larvae with dsRNA

One microgram of ds*Toll*, ds*Toll4*, ds*Toll6*, *dsToll6-1*, ds*Toll7,* and ds*Tollo* was injected into the 4th instar *A. chinensis* larvae, respectively. The larvae injected with ds*EGFP* were used as the control. Ten larvae were injected for each replicate, and triplicate was set for each treatment. Five larvae were sampled from each replicate 12 h and 48 h post-injection and flashfrozen in liquid nitrogen and then stored at −80°C for quantification of *Toll*, *Toll4*, *Toll6*, *Toll6-1*, *Toll7*, *Tollo*, *Defensin-1,* and *Defensin-2*.

### RT-qPCR for mRNA

Total cellular RNA was extracted using the Trizon method. cDNA was synthesized using MonAmp RTIII All-in-One Mix. Using cDNA as template, *β-actin* and *U6* as internal reference genes, *Toll*, *Toll4*, *Toll6*, *Toll6-1*, *Toll7*, *Tollo*, *Defensin-1*, *Defensin-2, ie1*, *vp39,* and *miR-8* transcripts were analyzed by RT-qPCR method. The sequence of primers is shown in Table 1. The relative transcription level of each gene was calculated by the 2^-ΔΔCT^ method.

### Statistical analysis

Statistical analyses were performed by GraphPad Prism 8 software. Data were shown as mean ± standard deviation (SD) from at least three independent experiments. The averages between the treatments and the controls were compared by *t*-test. *P* value < 0.05 is set for statistically significant (*P < 0.05, **P < 0.01, ***P < 0.001). The difference in gene expression between different instars and different tissues was analyzed by one-way ANOVA, and different letters were statistically significant (there were significant differences between a and b, b and c, c and d).

## RESULTS

### miR-8 and Toll receptors of *A. chinensis* are significantly responsive to AcMNPV-EGFP infection

**Fig 1:**
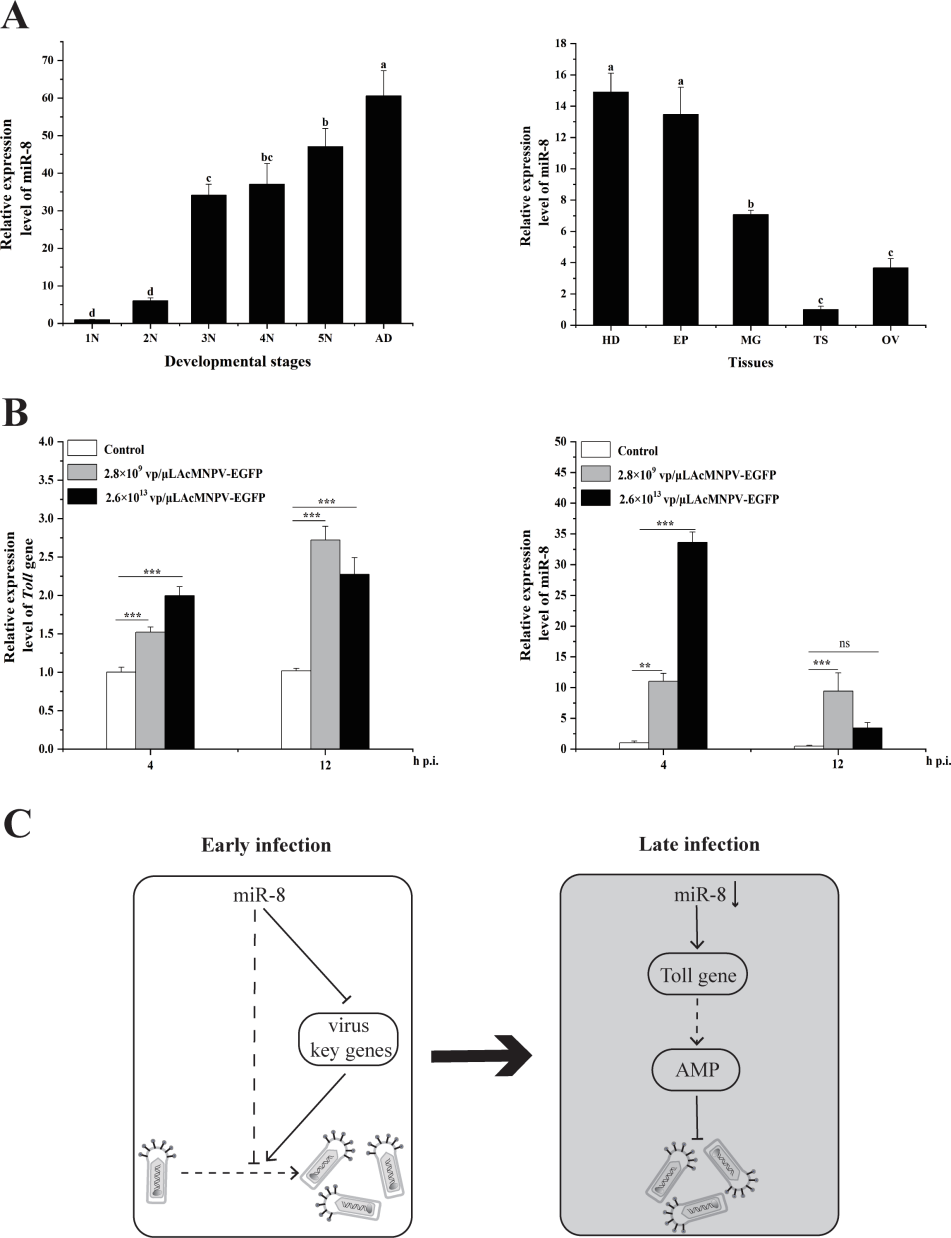
The spatiotemporal expression pattern of miR-8 in *A. chinensis* and the response of *Toll* gene and miR-8 to different concentrations of AcMNPV-EGFP infection. (**A**) Analysis of relative expression levels of miR-8 in different instars and in the epidermis, midgut, testis, and ovarian tissues of *A. chinensis*. (**B**) Response analysis of *Toll* gene and miR-8 of *A. chinensis* to different concentrations of AcMNPV-EGFP infection. (**C**) In the early stage of AcMNPV-EGFP infection, miR-8 targets the gene of AcMNPV-EGFP to inhibit virus proliferation. In the later stage of infection, miR-8 downregulates the *Toll* gene and increases the expression of AMPs to inhibit AcMNPV-EGFP proliferation.

The Toll signaling pathway is one of the essential signaling pathways regulating the expression of AMPs in insect innate immune response. AMPs have broad-spectrum resistance to bacteria, fungi, viruses, and other microorganisms and are one of the crucial effectors of humoral immunity. It has been reported that miR-8 in Drosophila targets multiple genes in the Toll signaling pathway, and the Toll receptor regulated by miR-8 is the key component of the Toll signaling pathway. In this study, RT-qPCR was used to detect the expression time of miR-8 from the first instar to the adult stage and the expression position in different tissues (Fig. 1A). The relative expression of miR-8 gradually increased with the development of *A. chinensis*. miR-8 may play an essential role in maintaining the stability of the physiological state of *A. chinensis*. Detecting the expression of miR-8 in different tissues such as head (HD), epidermis (EP), midgut (MG), testis (TS), and ovary (OV), the results showed that the expression of miR-8 was the lowest in testis, suggesting that miR-8 may also regulate the reproductive ability of male *A. chinensis* in addition to regulating innate immune response.

In this study, RT-qPCR was used to preliminarily analyze whether miR-8 and *Toll* genes have response and potential functions in AcMNPV-EGFP infection of *A. chinensis*. Our laboratory previously studied the improvement of the insecticidal activity by recombinant AcMNPV and designed a series of infection concentrations and detection times of wild viruses. In the pre-experiment, there was no significant change in miR-8 after 24 h of infection with 2.8 × 10^9^ vp/μL AcMNPV-EGFP. We speculate that the host response to a low concentration of virus is limited, and miR-8 is an important regulator, and its change should be completed in a short time. Therefore, we designed a set with higher infection concentration and shorter detection time. So, we used 2.8 × 10^9^ vp/μL low concentration AcMNPV-EGFP and 2.6 × 10^13^ vp/μL high concentration AcMNPV-EGFP to infect *A. chinensis* larvae and detected the expression changes of miR-8 and *Toll* genes by qPCR after infected 4 h and 12 h (Fig. 1B). The detection showed that the expression of *Toll* gene continued to increase after AcMNPV-EGFP infection, which may resist virus infection by promoting the expression of AMPs. However, the expression miR-8 increased at first and then decreased at 4 h and 12 h after infection with the recombinant virus. Moreover, the expression of miR-8 increased more obviously with infection AcMNPV-EGFP at high concentration. miR-8 in *B. mori* can downregulate the expression of *ie1* and *vp39* genes of BmNPV and inhibit the replication of the BmNPV genome. Therefore, we speculate that miR-8 targets the genes of AcMNPV-EGFP at the early stage of AcMNPV-EGFP infection to inhibit the virus proliferation; at the same time, miR-8 inhibits the proliferation of AcMNPV-EGFP by regulating the expression of AMPs through Toll signaling pathway at the late stage of infection (Fig. 1C).

### Identification and bioinformatics analysis of Toll family and AMPs in *A. chinensis*

**Fig 2:**
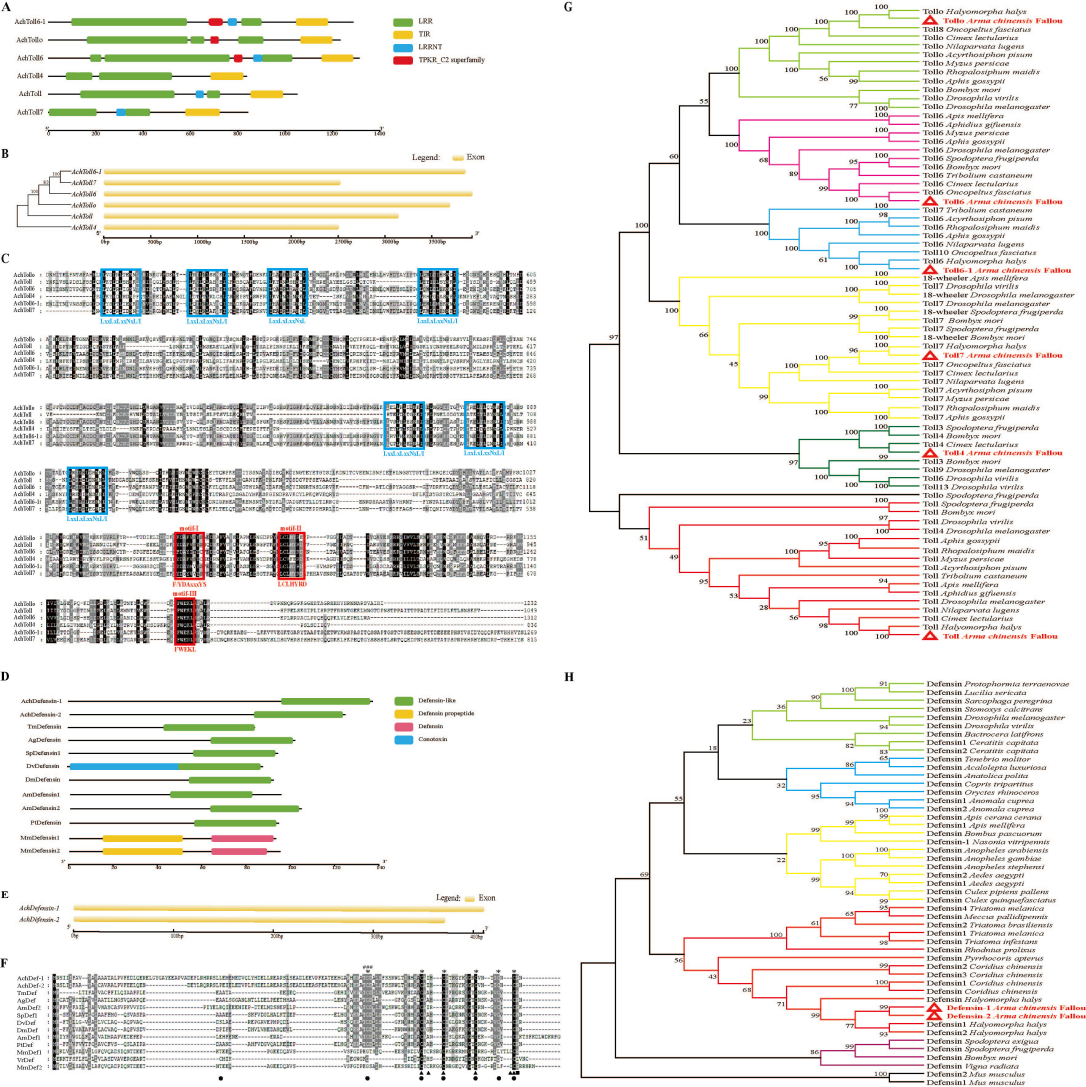
Bioinformatic analyses of the *Ach*Toll family and *Ach*Defensin. (**A**) The conserved domain of *Ach*Toll protein. The green bar represents the LRR domain, the blue bar represents the LRRNT structure domain, the yellow bar represents the TIR domain, and the red bar represents the TPKR_ C2 domain. (**B**) Exon intron organization in *AchToll* ORFs. Exons are displayed in light yellow rectangular boxes, and black lines between the boxes indicate introns. (**C**) Multiple sequence alignment of *Ach*Toll protein. The red box represents the three critical conserved motifs of the TIR domain, and the blue box represents the characteristic consistent sequence of the extracellular LRRs domain. (**D**) Conservative domain prediction of *Ach*Defensin and other typical insect and mammalian defensins. The green bar represents the defense-like domain, the blue bar represents the conotoxin domain, the yellow bar represents the defense propetide domain, and the red bar represents the defense domain. (**E**) The tissue distribution of exon-intron in *AchDefensin* ORFs. Exons are displayed in light yellow rectangular boxes, and black lines between the boxes indicate introns. (**F**) Multiple sequence alignment of two defensins subtypes from *A. chinensis* and typical defensins from other species. These species include seven insects: *Sarcophaga peregrina* (SpDef1, P18313), *Anopheles gambiae* (AgDef, Q17017), *Protophormia terraenovae* (PtDef, P10891), *Tenebrio molitor* (TmDef, Q27023), *Drosophila virilis* (DvDef, AHW49172), *Drosophila melanogaster* (DmDef, P36192), *Apis mellifera* (AmDef1, P17722) and a plant *Vigna radiata* (VrDef, AAR08912) and two mammalian species, *Mus musculus* (MmDef1, NP_034161 and MmDef2, NP_001182563). Asterisk indicates the position of cysteine in insect defensins, a solid triangle indicates the position of cysteine in mammalian defensins, and solid circles indicate the position of cysteine in plant defensins# Indicates threonine (T), cysteine (C), and cysteine (D), and the black box indicates arginine (R). (G) This tree is constructed using MEGA 11 based on the Neighbor-Wing algorithm (NJ). After 1,000 repetitions, the guided confidence value is displayed on the tree node. Insect species include *Arma chinensis* Fallou, *Halyomorpha halys*, *Oncopeltus fasciatus*, *Myzus persicae*, *Aphis gossypii*, *Apis mellifera*, *Aphis gossypii*, *Aphidius gifuensis*, *Rhopalosiphum maidis*, *Cimex lectularius*, *Tribolium castaneum*, *Nilaparvata lugens*, *Drosophila melanogaster*, *Drosophila virilis*, *Acyrthosiphon pisum*, *Spodoptera frugiperda,* and *Bombyx mori*. The Toll sequence of *A. chinensis* is marked in black font, and red font and is marked with a red triangle. (**H**) This tree is constructed using MEGA 11 based on the Neighbor-Wing algorithm (NJ). After 1,000 repetitions, the guided confidence value is displayed on the tree node. The *Ach*Defensin sequence is marked in red and bold font with a red triangle.

The research identified six *Toll* receptors and two *Defensin* subtypes genes: *AchToll*, *AchToll4*, *AchToll6-1*, *AchToll6*, *AchToll7*, *AchTollo*, *AchDefensin-1,* and *AchDefensin-2* from the transcriptome of *A. chinensis* (NCBI GenBank database: accession number SRX6830878), their physical and chemical properties are shown in Table 2. Conservative domain analysis showed that these deduced sequences were members of the Toll receptor family. They both contain a leucine-rich N-terminal domain (LRR) and a conserved TIR domain. The LRRNT domain is a leucine-rich repeat located in the cysteine-rich region. The TRKR_C2 superfamily refers to the Tyrosine-protein kinase receptor C2 Ig-like domain, which is related to β-neurotrophic factor interacts with each other, suggesting that *Ach*Toll6-1, *Ach*Toll6, and *Ach*Tollo receptors, containing this domain, may be involved in the neurotrophic process (Fig. 2A). The exon-intron organization of the six *Toll* genes of *A. chinensis* is shown in Fig. 2B. The comparison of genomic sequence and cDNA sequence shows that the six *AchToll* genes do not contain introns. Multiple sequence alignment identified three conserved motifs in the C-terminal TIR domain of the *Ach*Toll receptors: the initial F/YDAxxxYS motif, the intermediate LCLHYRD motif, and the final FWEKL motif. The LRRs domains are highly similar and contain the characteristic consensus sequence LxxLxLxxNxL/I (Fig. 2C).

**TABLE 2:** Physicochemical properties of *Ach*Toll and *Ach*Defensin

Protein	ORF length (bp)	Protein sequence (aa)	Molecular weight (kDa)	Isoelectric points	Leucine ratio (%)	Hydropathicity	Instability index
*Ach*Toll	3,150	1,049	118.5	6.17	15.4% (162)	−0.096	36.69
*Ach*Toll4	2,511	836	95.88	6.22	14.5% (121)	−0.033	47.04
*Ach*Toll6-1	3,861	1,286	145.38	5.41	14.5% (183)	−0.192	42.13
*Ach*Toll6	3,939	1,312	149.83	6.21	14.6% (191)	−0.158	48.32
*Ach*Toll7	2,532	842	97.58	6.33	11.4% (96)	−0.358	50.22
*Ach*Tollo	3,699	1,232	140.47	5.53	15.1% (186)	−0.144	38.09
*Ach*Defensin-1	414	137	15.39	5.09	9.5% (13)	−0.207	56.32
*Ach*Defensin-2	375	124	13.88	5.18	10.5% (13)	−0.190	65.37
*Ach*Defensin^*a*^	132	43	4.634	8.91	4.7% (2)	0.033	28.13

^*a*^ *Ach*Defensin is a mature peptide of *Ach*Defensin-1 and *Ach*Defensin-2.

The conserved domains of *Ach*Defensin, other insect defensins, and two animal defensins were analyzed. The results showed that insect defensins contained defensin-like domains, indicating that the identified genes were *Defensin* genes (Fig. 2D). In contrast, animal defensins had two conserved domains: defensin propeptide domain and mammalian defensin domain. The exon-intron organization of the two *Defensin* subtype genes of *A. chinensis* is shown in Fig. 2E. The comparison of genome and cDNA sequences shows that both *Defensin* genes contain only one exon. Multiple sequence alignment results showed that insect and mammalian defensins had six conserved cysteine residues, while plant defensins had eight cysteine residues. The position of cysteine between insect and plant defensins is similar to that of mammalian defensins but is very different (Fig. 2F). In addition, two typical characteristics of most insect defensins mature peptides are threonine residues, cysteine residues, and alanine residues (-TCD-) at the N-terminal and arginine residues (-R-) at the C-terminal.

The Toll proteins of insects are highly similar (Fig. 2G). Phylogenetic analysis of Toll proteins in *A. chinensis* and other insects showed that the six *Ach*Toll receptors were divided into six branches. Each branch was clustered with the corresponding Toll receptors of other insects, which was highly similar to the Toll receptors of other Hemiptera insects. The phylogenetic tree shows that insect defensins and plant defensins are divided into one branch, and mammalian defensins are used as outgroups (Fig. 2H). Defensins of Diptera insects other than the mosquito family are clustered into one branch, defensins of Coleoptera insects are clustered into one branch, and defensins from mosquitoes and Hymenoptera insects belong to another branch. Hemiptera insect defensins containing *Ach*Defensin are clustered into one branch. Lepidoptera insects and a plant defensin clustered into a branch, suggesting that there may be horizontal gene transfer between the plant and Lepidoptera insects. In the process of evolution, *Ach*Defensin-1 and *Ach*Defensin-2 have the closest homology with the defensins of *Halyomorpha halys*.

### Identification of the temporal and spatial expression profiles of *Toll* family and *Defensins* in *A. chinensis*

**Fig 3:**
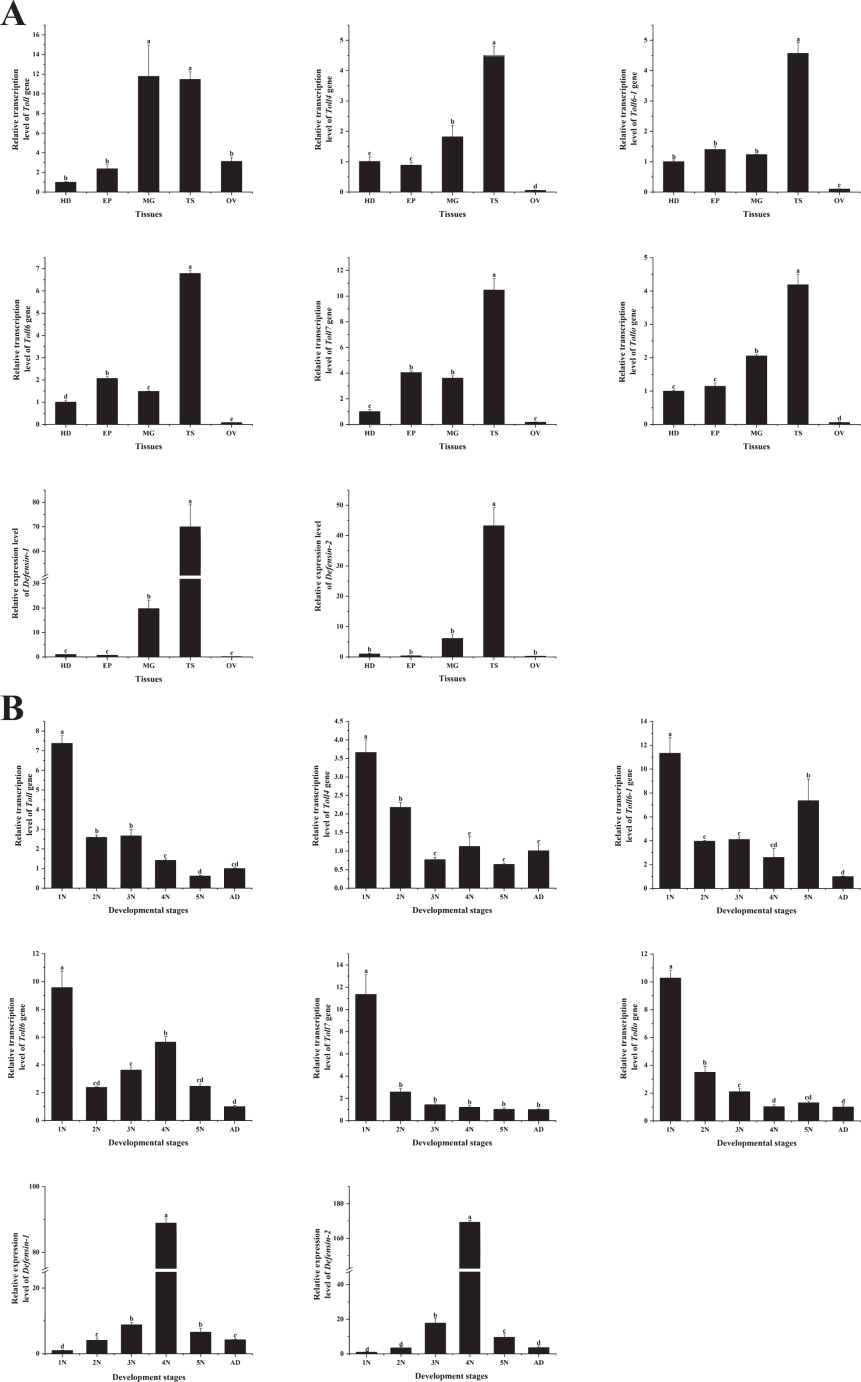
Relative expression levels of the *Toll* family and two *Defensin* subtypes in different tissues and developmental stages of *A. chinensis*. (**A**) Expression analysis of the *Toll* family and two *Defensin* genes in the head (HD), epidermis (EP), midgut (MG), testis (TS), and ovarian (OV) tissues. (**B**) The relative expression levels of the *Toll* family and two *Defensin* genes in different age stages of *A. chinensis.* N indicates instar, and AD indicates adult.

Toll family in insects regulates innate immune response and plays a regulatory role in insect development. In the early stage of this study, six *Toll* and two *Defensin* genes were identified by analyzing the transcriptome of *A. chinensis*, and the temporal and spatial expression patterns of these genes were identified in different instars and tissues including the head (HD), epidermis (EP), midgut (MG), testis (TS), and ovary (OV) (Fig. 3). The expression levels of *Toll* and *Defensin* genes in different tissues were detected, and they were found to be highly expressed in testis (Fig. 3A), suggesting that *Toll* family and *Defensin* may play a role in the development of the spermatozoa and sperm formation. Second, these genes are highly expressed in the midgut, which may play a major role in the signal transduction of the immune pathway. In general, the six *Toll* genes were highly expressed at the first instar and lowly expressed at the adult stage (Fig. 3B), indicating that these genes may be involved in the early development of *A. chinensis*. However, the *Defensin* gene was highly expressed in the fourth instar, suggesting that the fourth instar, which developed wing buds in the paurometabola insect, was susceptible to microbial infection and death during metamorphosis, so the *Defensin* genes were significantly highly expressed at this age.

### miR-8 regulates the expression of six *Toll* and two *Defensin* genes

**Fig 4:**
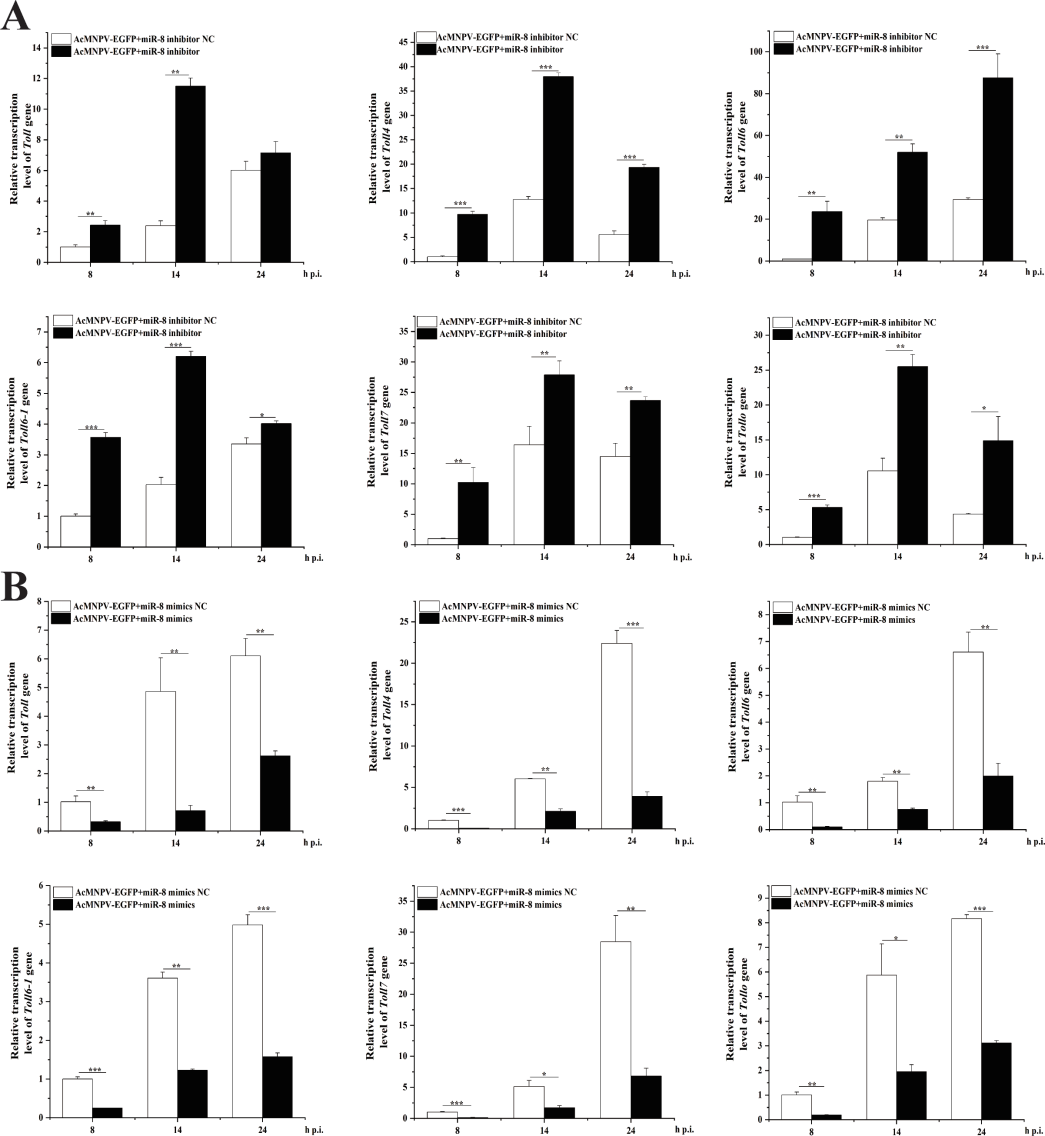
miR-8 regulates the expression of *Toll* family genes in *A. chinensis*. (**A**) The miR-8 inhibitor upregulates the expression of the *AchToll* family genes. (**B**) miR-8 mimics downregulates the expression of the *AchToll* family genes.

Subsequently, to determine whether miR-8 regulates the expression of *Toll* genes in *A. chinensis*, the expression levels of six *Toll* genes were detected by RT-qPCR after injection of miR-8 mimics and inhibitor (Fig. 4). The results showed that the expression of these *Toll* genes was upregulated at 8 h, 14 h, and 24 h after miR-8 inhibitor was used to inhibit the role of miR-8 (Fig. 4A). miR-8 mimics were used to increase the expression of miR-8, and the expression of these genes was inhibited (Fig. 4B). These results suggest that miR-8 is, indeed, a negative regulator of the Toll receptor in the Toll signaling pathway and other Toll receptors in the Toll family.

### miR-8 regulates the expression of Defensin through Toll receptors and targets *ie1* and *vp39* genes to regulate the resistance of *A. chinensis* together to AcMNPV-EGFP infection

**Fig 5:**
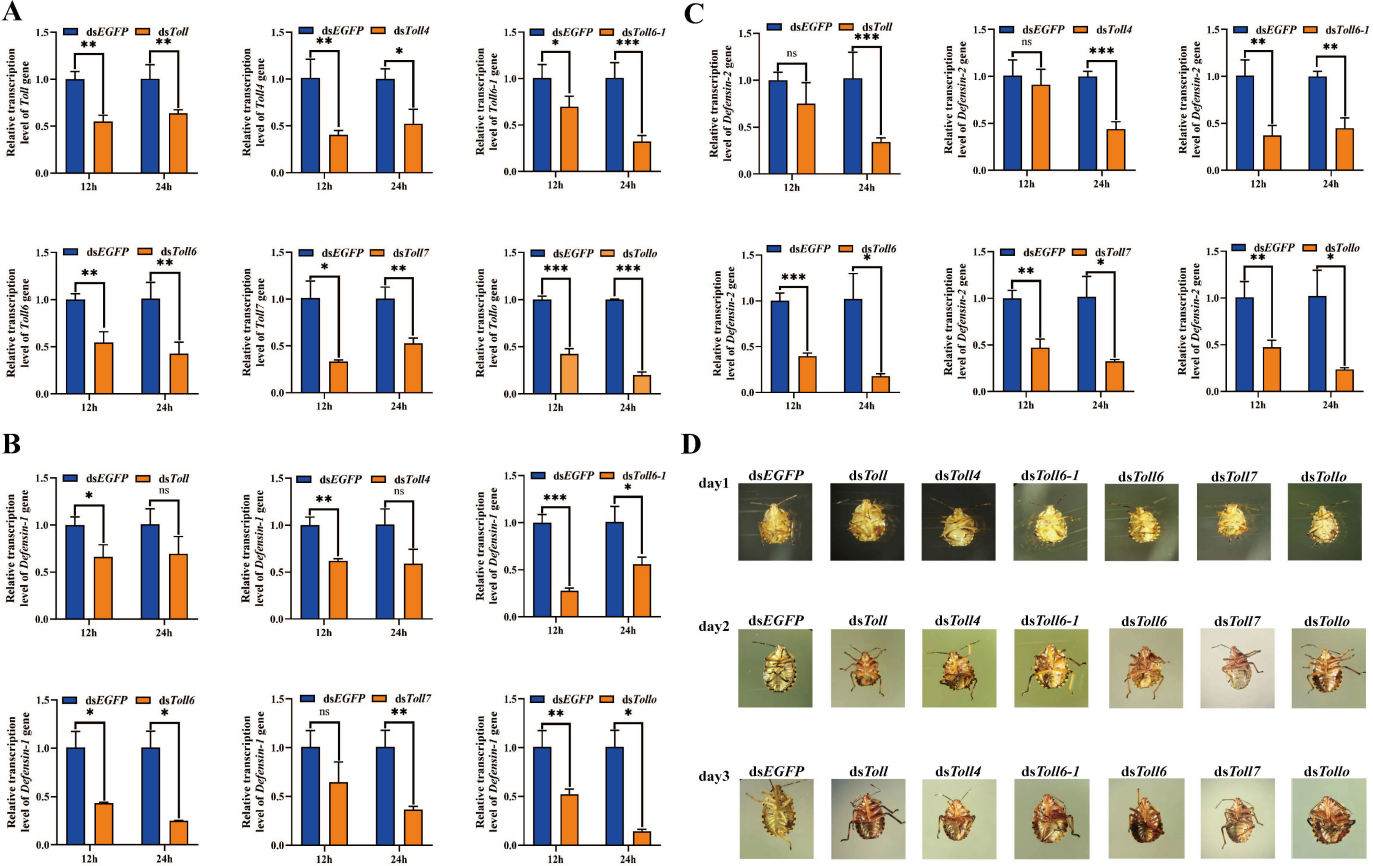
RNAi interferes with *Toll* family genes in *A. chinensis*. (**A**) The effect of qPCR detection on RNAi interference in *Toll* family gene expression. (**B**) Changes in the expression level of *Ach*Defensin-1 after interfering with the *Toll* family genes at 12 h and 24 h. (**C**) Changes in the expression level of *AchDefensin-2* after interfering with the *Toll* family genes at 12 h and 24 h. (**D**) Injecting ds*EGFP*, ds*Toll*, ds*Toll4*, ds*Toll6*, ds*Toll6-1*, ds*Toll7*, and ds*Tollo* into the fourth instar larvae of *A. chinensis*, within 3 days the larvae in the other experimental groups died in large numbers, turned red on the surface, and gradually liquefied and shrunk compared to the group injected with ds*EGFP* after injecting dsRNA.

**Fig 6:**
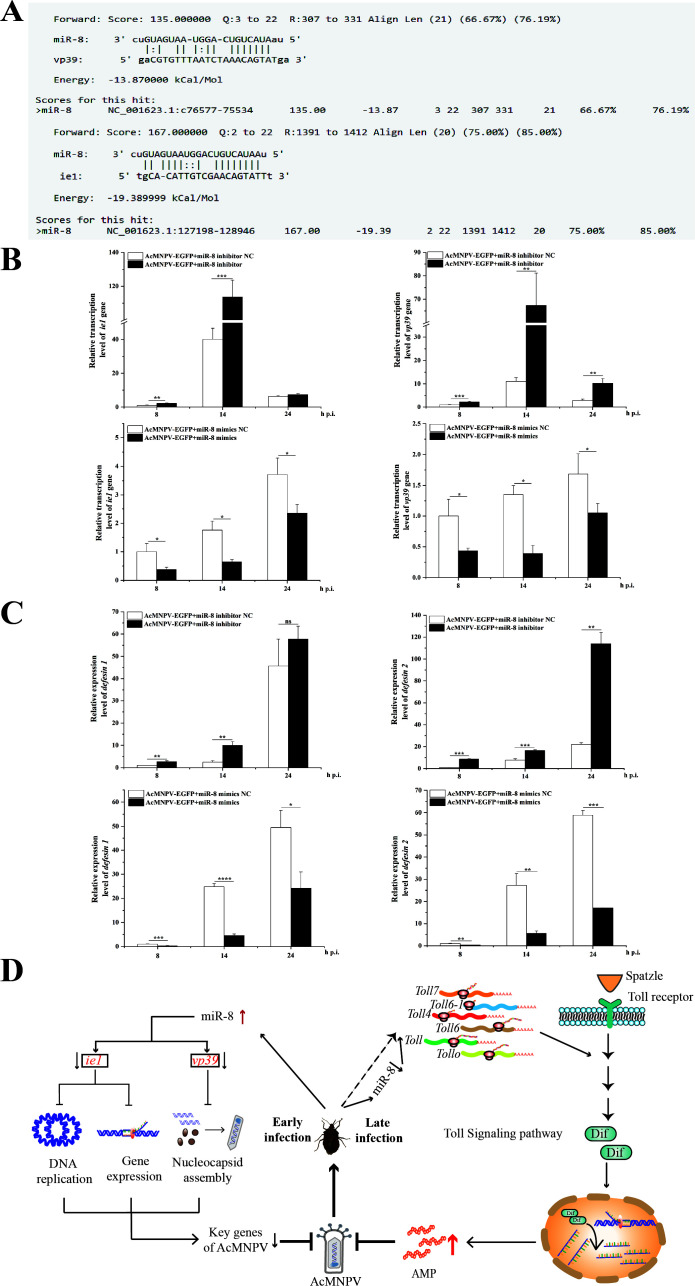
miR-8 downregulates the expression of two *Defensin* genes in *A. chinensis* and targets the *ie1* and *vp39* genes in AcMNPV-EGFP. (**A**) miRanda predicts that the *ie1* and *vp39* genes in AcMNPV-EGFP are targets of miR-8. (**B**) miR-8 targets the *ie1* and *vp39* genes of AcMNPV-EGFP. (**C**) miR-8 inhibits the expression of two *Defensin* genes. (D) In the early stage of AcMNPV-EGFP infection, miR-8 inhibits the proliferation of AcMNPV-EGFP by downregulating the expression of *ie1* and *vp39* genes. In the late stage, miR-8 upregulates the expression of *Defensin* by upregulating the expression of *Toll* to resist AcMNPV-EGFP infection.

We identified the Toll receptor in the Toll signaling pathway regulated by miR-8 and identified two *Defensin* genes from the transcriptome of *A. chinensis*. RNAi detected the regulation of the *Toll* family on *Defensin* expression. The results showed that the *Toll* family expression was significantly interfered, and the expression of two kinds of *Defensin* genes was downregulated at 12 h and 24 h after injection of dsRNA of *Toll* family genes (Fig. 5). At the same time, interfering with the *Toll* family resulted in a large number of deaths of *A. chinensis* larvae in a short time. After injection of the *Toll* family dsRNA, the movement of *A. chinensis* larvae became slow, their body surface turned red, and their body gradually liquefied and shrunk, indicating that the Toll family is crucial to the survival of the *A. chinensis* larvae (Fig. 5D). In addition, miRanda predicted that the early gene *ie1* and late gene *vp39* of AcMNPV were the target genes of miR-8 (Fig. 6A). To further study the antivirus immune mechanism of miR-8 in *A. chinensis*, miR-8 mimics and inhibitor were used to detect the effect of miR-8 on the expression of *Defensin* in *A. chinensis* and *ie1* and *vp39* genes in AcMNPV-EGFP. RT-qPCR results showed that the expressions of the two *Defensin*, *ie1,* and *vp39* genes were upregulated after inhibiting the role of miR-8. In contrast, the expression of these genes was inhibited by increasing the expression of miR-8 (Fig. 6B and C). Combined with the trend that the relative expression of miR-8 increased first and then decreased after AcMNPV-EGFP infected in *A. chinensis*, the above studies showed that miR-8 inhibited the proliferation of AcMNPV-EGFP by downregulating the expression of *ie1* and *vp39* genes in the early stage of AcMNPV-EGFP infection and then increased the expression of *Defensin* by upregulating the expression of Toll family to resist AcMNPV-EGFP infection.

## DISCUSSION

The AcMNPV we used in this study is not a specific virus for *A. chinensis*. Although AcMNPV is a unique virus of Lepidoptera, many studies have shown that AcMNPV can infect non-host insects and replicate and express genes in non-host insects (5–7). Combined with our experimental results, we determined that high concentrations of AcMNPV could infect the larvae of *A. chinensis* and express genes. As a biological insecticide, baculovirus has a good potential for application in pest control in agriculture and forestry. The use of natural enemies is also a green means of biological control. To better control pests, natural enemy insects and other pesticides should be reasonably combined to avoid pesticides that affect the reproduction and development of natural enemy insects. In this study, we demonstrated that *A. chinensis* was immune responsive to the Lepidopteran insecticide AcMNPV. *A. chinensis* was not sensitive to AcMNPV, suggesting that *A. chinensis* might serve as an intermediate vector to promote the spread of AcMNPV. Even though AcMNPV is not lethal to *A. chinensis* at non-high concentrations, we still need to investigate its effects on other aspects of insect physiology, including lifespan, mating, and reproduction. In addition, this study only examined the effects of AcMNPV on *A. chinensis* in the laboratory but not in the field. In the future, we can study the specific effects of residual AcMNPV on natural enemy insects in natural environments and find the middle point of maximizing the use of biological control and insecticide control combined with the pest control effect of AcMNPV.

miR-8 is a highly conserved miRNA that widely exists in bilaterally symmetrical animals. In *Drosophila melanogaster*, miR-8 coordinates the immune response by regulating the activities of Toll, Notch, and EGFR pathways (27). This study found that miR-8 had a significant response in the process of AcMNPV-EGFP infecting *A. chinensis* larvae, and the expression of miR-8 increased significantly in the early stage of AcMNPV-EGFP infection and then decreased significantly. To further reveal the molecular mechanism of miR-8 in the antiviral immune response of *A. chinensis*, through literature review, we found the *Toll* gene of *A. chinensis*, the transactivator *ie1* gene, and the major capsid protein *vp39* gene of AcMNPV-EGFP may be the target of miR-8. After that, miRanda predicted that *ie1* and *vp39* genes were the target genes of miR-8, and the injection of miR-8 mimics and miR-8 inhibitor proved that miR-8 was the negative regulator of the *Toll* receptors in the important immune pathway of *A. chinensis*, and *ie1* and *vp39* of AcMNPV-EGFP. We also speculate that miR-8 targets the AcMNPV-EGFP genes at the early stage of AcMNPV-EGFP infection to inhibit the proliferation of the virus, while miR-8 inhibits the proliferation of AcMNPV-EGFP by regulating the Toll signaling pathway to increase the expression of AMPs at the late stage of infection.

Toll receptor is the core component of the Toll signaling pathway, which plays a key role in insect embryonic development and innate immunity (28). There is the Toll family in insects. Different Toll receptors have different functions and regulate immune, developmental, and neurotrophic processes in insects (28–31). We identified six *Toll* genes of *A. chinensis*, less than the number of *Toll*-related genes identified in other insects (32). Multiple sequence alignment results showed that three typically conserved motifs of the Toll family were found in the six *Ach*Toll amino acid sequences (33, 34). Different domains may play different functions. The TIR domain is the key site that interacts with the cytoplasmic adaptor protein MyD88 to achieve intracellular signal transduction, and the LRR domain may participate in the interaction between proteins (35). These results suggest that the six *AchToll* genes are members of the Toll family, and their functions may be conserved in the direct homologs of other species. *AchToll* genes are highly expressed at some ages, indicating that these genes may be involved in various biological processes. We found that six *AchToll* genes were highly expressed in first instar, which may be due to the involvement of these Toll receptors in the early development of *A. chinensis. AchToll6* and *AchToll6-1* were highly expressed at the fourth and fifth instar, respectively. A large amount of feeding and wing bud development of the fourth and fifth instar larvae may significantly increase the probability of exposure to various pathogens. Insects may be responded to by increasing the expression levels of *Toll6* and *Toll6-1*. The tissue-specific expression analysis of the *AchToll* genes was the highest in testis and midgut, indicating that *Toll* genes may participate in testis development and sperm formation and play a role in the signal transduction of immune response. Toll receptors in Drosophila play an essential role in X virus infection (36), and members of the Toll family have potential antiviral functions. During AcMNPV-EGFP infection, we detected that *Toll* gene expression was upregulated, suggesting that the Toll family may play an important role in the antiviral immunity of *A. chinensis* through the Toll signaling pathway.

AMPs, downstream factors of the Toll signaling pathway, are essential effectors in insect innate immune response. We identified two subtypes of defensins, *AchDefensin-1* and *AchDefensin-2*, from the transcriptome of *A. chinensis*. As the main member of insect AMPs, defensins are a class of small peptides rich in cysteine and mostly with cations. They have intense activity against Gram-positive bacteria and weak activity against Gram-negative bacteria and fungi. The mature peptide of *Ach*Defensin has similar sequence and structural characteristics to most insect defensins. The motif of *Ach*Defensin is C-×16-C-×3-C-×9-C-×4-C-×1 C and is consistent with the typical one of a defensin (37). In addition, *Ach*Defensins are also typical cationic peptides, and the pI of its mature peptide is 8.91, which may produce an antibacterial effect through mutual attraction. In the developmental stage of *A. chinensis*, both *AchDefensin* isoforms were highly expressed in the fourth instar. This may be because *A. chinensis* larvae are more susceptible to pathogen infection during the development of wing buds at the fourth instar. Therefore, *A. chinensis* larvae improve their ability to resist pathogen invasion by increasing the expression level of *AchDefensin*. Two *AchDefensin* genes were highly expressed in the midgut and testis in different tissues. It suggested that *AchDefensin* may resist pathogen infection and play a role in testis development and sperm formation. We found that inhibition of the *AchToll* family can downregulate the expression of *AchDefensin*, and miR-8 can negatively regulate the expression of *AchToll* and *AchDefensin*. Therefore, *AchDefensin* may have not only antibacterial activity but also antiviral activity. As a class of small cationic peptides, AMPs are easy to obtain and chemically modify and are not easy to produce drug resistance in medical applications. Therefore, developing and improving AMP is a direction and subject with great potential and practical value. *Ach*Defensin is one of the common AMPs in insects and may provide a new drug for human medicine. In the future, the inhibitory efficiency of *Ach*Defensin against various pathogens can be identified, and *Ach*Defensin can be modified to improve its efficacy.

In conclusion, this study emphasized that miR-8 played an important role in antiviral immunity by regulating AcMNPV-EGFP essential genes and *A. chinensis* genes. Six Toll receptors and two Defensin subtypes were also identified in *A. chinensis*, which laid the foundation for studying the Toll family regulating the growth, development, immunity, and reproduction of *A. chinensis* and promoting the understanding of the immune regulation mechanism of *A. chinensis*. This study revealed that AcMNPV activated the immune response of natural enemy insects by regulating the miR-8-Toll-Defensin axis, providing molecular basis for optimizing the joint application of viral insecticides and natural enemies.

## Data Availability

The NCBI gene accession numbers of *ie1, vp39, AchToll, AchToll4, AchToll6, AchToll6-1, AchToll7, AchTollo, AchDefensin-1,* and *AchDefensin-2,* respectively, are 1403980, 1403922, PP263627, PP263628, PP263629, PP263630, PP263631, PP263632, PP263633, and PP263634.
